# Plant volatiles and priority effects interactively determined initial community assembly of arthropods on multiple willow species

**DOI:** 10.1002/ece3.10270

**Published:** 2023-07-19

**Authors:** Kinuyo Yoneya, Takeshi Miki, Noboru Katayama

**Affiliations:** ^1^ Faculty of Agriculture Kindai University Nara Japan; ^2^ Center for Biodiversity Science Ryukoku University Otsu Japan; ^3^ Faculty of Advanced Science and Technology Ryukoku University Otsu Japan; ^4^ General Education Faculty of Commerce Otaru University of Commerce Otaru Japan

**Keywords:** community assembly, plant–animal interaction, plant‐arthropod feedback

## Abstract

Plant traits, which are often species specific, can serve as environmental filtering for community assembly on plants. At the same time, the species identity of the initially colonizing arthropods would vary between plant individuals, which would subsequently influence colonizing arthropods and community development in the later stages. However, it remains unclear whether interindividual divergence due to priority effects is equally important as plant trait‐specific environmental filtering in the initial stages. In this study, we propose that plant volatile organic compounds (PVOCs) may play a crucial role as an environmental filter in the initial stages of community assembly, which can prevent the community assembly process from being purely stochastic. To test this hypothesis, we conducted short term but highly frequent monitoring (19 observations over 9 days) of arthropod community assembly on intact individuals of six willow species in a common garden. PVOC compositions were analyzed before starting the experiment and compared with arthropod compositions occurring on Days 1–2 of the experiment (*earliest colonizer community*) and those occurring on Days 8–9 of the experiment (*subsequent colonizer community*). Unintentionally, deer herbivory also occurred at night of Day 2. Distance‐based statistics demonstrated that PVOC compositions were plant species specific, but neither the earliest colonizer nor the subsequent colonizer community composition could be explained by plant species identity. Rather, Procrustes analysis showed that both the PVOC composition and that of the earliest colonizer community can be used to explain the subsequent colonizer community. In addition, the linkage between PVOCs and the subsequent colonizer community was stronger on individuals with deer herbivory. These findings indicate that PVOCs have widespread effects on initial community assembly, as well as priority effects brought on by stochastic immigration, and that plant species identity only has weak and indirect effects on the actual composition of the community.

## Graphical abstract

We elucidated the roles of plant volatile organic compounds (PVOCs) and priority effects in initial stages of the arthropod community assembly on six willow species. Despite the clear species‐specificity of PVOC composition, arthropod community assembly was better explained by interindividual variations of PVOC composition and the stochastically determined earliest colonizer community composition (structured in 2 days).

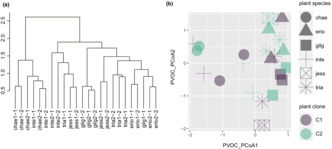

## Abstract (ja)

植物の形質は多くの場合種特異的であり，植物上の群集集合における環境フィルタリングとして機能すると考えられる.同時に，最初に植物上に移入してくる節足動物は植物個体間で種が異なるであろうことが予想される.したがって，どの種が最初に移入してくるかは，その後に移入してくる節足動物の種類と形成される群集の発展過程に影響すると考えられる.しかし，群集集合の最初期においても，先住効果によって引き起こされる植物個体間での群集組成の多様化が，植物種特異的な環境フィルタリングと同程度に顕著であるかは未解明なままである.本研究では，植物の揮発性有機化合物(PVOC)が最初期の群集集合において環境フィルタリングとして機能し，節足動物の移入が完全に確率的に決まるのではないとの仮説を提案する.この仮説を検証するため，実験圃場において6種の健全ヤナギ個体上で，節足動物の群集集合を短期間・高頻度でモニタリングした(9日間で19の観測).実験開始前にPVOC組成を分析し，実験開始1‐2日目に形成された節足動物組成(「最初期移入者」群集)と実験開始8‐9日目に形成された節足動物組成(「二次移入者」群集)とを比較した.また，予期せず2日目の夜間にシカによる食害も発生した.距離ベースの多変量解析の結果，植物個体間のPVOC組成の違いは，ヤナギ樹種の違いによって説明された.一方，最初期移入者群集と二次移入者群集の植物個体間での組成の違いはどちらもヤナギ樹種の違いでは説明できなかった.むしろ，Procrustes解析により，PVOC組成と最初期移入者群集の組成の両方が，二次移入群集の組成を説明できることが示された。また，Procrustes解析の残差に注目すると，PVOCと二次移入群集との関連性は，シカの食害が起きた個体ほど強いことが示された.これらの結果は，非決定論的な初期移入が発端となる先住者効果に加えて，PVOCは初期の群集集合に広く影響を与えたことを示す一方，植物種間の違いは群集集合に対して弱く間接的な影響しか持たないことを示唆している.

## 1 INTRODUCTION

Arthropod communities on plants are one of the model systems for community assembly research, with dynamics driven by interactions between arthropod populations and plant traits (acting as environments; Utsumi et al., 2010). Plant traits are often species specific (Hopkins et al., 2017; Lehrman et al., 2012; Moreira et al., 2014) and genotype specific (Barbour et al., 2015; Lehrman et al., 2013), which act as environmental filtering (Chen et al., 2017; Götzenberger et al., 2012; Zobel, 1997) for arthropod community assembly. This results in arthropod community composition depending on the plant species and genotype identity (Hochwender & Fritz, 2004). At the same time, induced responses such as induced resistance and compensatory regrowth are herbivore‐species‐specific and deterministic (Kessler & Halitschke, 2007; Van Zandt & Agrawal, 2004a), which in turn affect the subsequent community assembly (Stam et al., 2018; Van Zandt & Agrawal, 2004b), forming feedback between plant traits and arthropods (i.e., plant–arthropod feedback, Utsumi et al., 2010; Yoneya et al., 2023). When such feedback is initiated by the earliest colonizers on a plant whose identity can vary stochastically (Bacca et al., 2021; Shinohara & Yoshida, 2021), it results in a priority effect (Chase, 2003; Fukami, 2015) because of the combination of stochastic and deterministic processes (Chase, 2010), which contribute to the divergence of community composition even within a plant population.

Plant volatile organic compounds (PVOCs), a key plant trait, influence the behavior of arthropods, including both herbivores and their natural enemies, by acting as attractants and repellents (Yoneya & Miki, 2015 and references therein). PVOCs affect herbivore communities and their predator and parasitoid populations (Fatouros et al., 2012; Karban & Shiojiri, 2009; Morrell & Kessler, 2017; Xiao et al., 2012). Intriguingly, PVOC compositions are not only plant species‐specific (Peacock et al., 2001; Yoneya et al., 2012), but also depend on the identity of herbivore species (Mann et al., 2021) and co‐occurring plant species (Grof‐Tisza et al., 2021; Yoneya et al., 2012) especially when induced by herbivory. For example, the composition of uninfested willow PVOCs differs significantly among cooccurring seven willow species, whereas the similarity of PVOCs induced by herbivory of the common leaf beetle species is high in some of these seven species and attracts its natural enemy predator more effectively than other willow species (Yoneya et al., 2012).

The current experimental approaches, which primarily focus on single or limited tri‐trophic food chains within arthropod communities and rely on choice tests for individual species, are ineffective in understanding the roles of PVOCs in highly diverse arthropod communities. This limitation arises from the difficulty of conducting tests for every arthropod species, particularly minor ones. Moreover, the community composition cannot be explained by the superposition of each species' preference to PVOCs, as arthropod species interact with each other in various ways, including competition and prey–predator interactions (Yoneya & Miki, 2015). Nevertheless, very few studies (Zu et al., 2020) have investigated the linkage between PVOCs and diverse arthropod communities. At the same time, the divergence of arthropod communities between plant individuals due to priority effects has also received increasing attention (Stam et al., 2018; Utsumi, 2015; Van Zandt & Agrawal, 2004b; Yoneya et al., 2023). Previous studies have addressed the role of initial stage communities in shaping later stages of community development by experimentally manipulating either the initial herbivore colonizers (Stam et al., 2018; Utsumi, 2015; Van Zandt & Agrawal, 2004b) or the plant initial conditions, including exposure to herbivore‐induced PVOCs (Yoneya et al., 2023), while maintaining other environmental conditions homogeneous. The manipulation of initial colonizers in a homogeneous environment has been widely applied to microbial (Dickie et al., 2012; Toju et al., 2018) and plant (Fukami et al., 2005) communities. Consequently, the structuring of initial stage communities by dispersal and interspecific interactions in a heterogeneous environment remains unclear. Specifically, it remains underexplored how interindividual divergence resulting from priority effects interacts with plant trait‐specific environmental filtering during the initial stages of community assembly, encompassing both stochastic and deterministic processes (Chase, 2010). In this study, we propose that PVOCs may play a crucial role as an environmental filter in the initial stages of community assembly, which can prevent the community assembly process from being purely stochastic. PVOCs can alter the behaviors of both herbivores and their natural enemies in a predictable way, affecting the mortality of herbivores as well as immigration and emigration of herbivores and carnivores, thereby acting as the environmental filter.

The overarching objective of this research is to contribute to a better understanding of the mechanisms underlying the initial stages of community assembly by investigating the proposed role of PVOCs as the environmental filter. We conducted short term (9 days from the establishment) but highly frequent (two–three times per day) monitoring of arthropod community assembly on four intact potted individuals (two clones × two individuals per clone) from each of the six willow species in a common garden. We sampled the headspace around the potted plant to analyze PVOC compositions from all individuals (24 in total) before starting the community assembly experiment and compared them with arthropod compositions occurring on Days 1–2 of the experiment (hereinafter, termed the *earliest colonizer community*) and those occurring on Days 8–9 of the experiment (termed the *subsequent colonizer community*). Deer herbivory occurred unintentionally during the second night of the community assembly experiment; therefore, we investigated its influence on the colonizer communities.

With this monitoring, we specifically aimed to test the overall connection between PVOCs, arthropod communities, and plant species identity, and to examine how the strength of the connection is determined. To achieve this, we combined distance‐based (PERMANOVA and Mantel test) and raw‐based (Procrustes analysis; Lisboa et al., 2014; Peres‐Neto & Jackson, 2001) approaches. Procrustes analysis, which is comparable to the Mantel test but offers greater statistical power for detecting the overall linkage between matrices (Peres‐Neto & Jackson, 2001), not only allows us to assess the overall linkage but also provides a means to evaluate the strength of linkage for individual observations. Meanwhile, distance‐based approaches have long been employed in community assembly studies (Anderson et al., 2011). By incorporating raw‐based approaches alongside distance‐based approaches, we can further untangle the complex relationships between PVOCs and arthropod communities. We developed three hypotheses that were not mutually exclusive to explore different factors influencing community assembly. First, we hypothesized that both plant species identity and PVOCs contribute to the community assembly. This hypothesis allowed us to examine whether PVOCs and other plant traits act as environmental filters. Plant species identity served as a proxy for unmeasured plant species‐specific traits. Second, we hypothesized that the earliest colonizer composition also affects how the community is assembled later. This hypothesis aimed to investigate the significance of priority effects in the initial stages of community assembly, in addition to environmental filtering. Finally, we hypothesized that large herbivores can influence community assembly. Our monitoring design primarily focused on testing the first and second hypotheses, while the including of the third hypothesis aimed to provide a possible explanation for any unintended impacts of deer herbivory (Fraser et al., 2018).

## 2 MATERIALS AND METHODS

### 2.1 Plants

Six willow species were used: *Salix integra* (**Inte**)(*Inukori yanagi*), *Salix eriocarpa* (**Erio**)(*Ja yanagi*), *Salix gilgiana* (**Gilg**)[or *Salix miyabeana* (*Kawa yanagi*)], *Salix serissaefolia* (**Jess**)[or *S. jessoensis* Seemen subsp. *serissaefolia* (Kimura), Ohashi, 2000] (*Kogome yanagi*), *Salix chaenomeloides* (**Chae**)(*Maruba yanagi*), and *Salix triandra* (**Tria**)(*Tachi yanagi*). The abbreviations in the first brackets after scientific names are used in the following text and figures. The words in the second brackets are the corresponding Japanese names of the willow species, which would help avoid confusion related to the synonyms in willow taxonomy, since these are more stable and have fewer synonyms (Ohashi, 2000). The natural populations of these six species, as well as *S. gracilistyla* (*Neko yanagi*), occur sympatrically in a floodplain as the predominant woody plants along the Yasu River (35°03'22.5"N, 136°00'45.2"E) in Siga Prefecture, central Japan. Willow stems from multiple clones of each species were collected from these populations and cultivated in an experimental willow garden at the Center for Ecological Research (CER) of Kyoto University (34°58′17″N, 135°57′32″E, Otsu, Japan, 10.6 km south of the Yasu River) since 2002.

We cut and collected 1–2‐year‐old branches of willows (two branches per clone × two clones × six species) from the willow garden in the CER on May 13, 2016. After keeping their basal section in water for 10 days until root emergence, we potted them individually in soil (Takii potting soil, Takii & Co., Ltd.) in pots (diameter 9 cm, height 7.6 cm) and supplied them with fertilizer (Hyponex (N–P–K = 6–10–5); HYPONeX) every 2 weeks. Potted plants were maintained in a greenhouse under a long‐day photoperiod (18 L: 6 D) at 25 ± 2°C and 50%–70% RH until the newly emerged shoots were approximately 15–20 cm in height with approximately 20 leaves. We used four potted plants for each willow species (two clones × two replicates per clone). These potted plants were used to collect plant volatiles and then located randomly in three lines of eight pots at 2‐m interval in the experimental garden next to the willow garden in CER on June 21, 2016. It appears that replicates within each clone are pseudo‐replicates. However, the high vegetative reproduction ability of floodplain species of willows (Radtke et al., 2012) can result in the dominance of a single clone in multiple populations along a single river (Beismann et al., 1997), leading to neighboring willow individuals in a short distance being genetically identical in natural environments. Therefore, including multiple individuals from a single clone as independent replicates was reasonable to investigate the roles of stochastic colonization and priority effects in an identical environment (identical genetical background), as well as environmental filtering (from different plant clones and species) in community assembly processes. Differences resulting from plant clones were not our main focus but treated as a nested factor within plant species in the statistical analyses.

### 2.2 PVOC characterization

The volatiles were collected from each potted plant in a climate‐controlled room. A hexane solution of tridecane (0.5 mg/mL) impregnated into a piece of filter paper (1 cm^2^) was used as the internal standard. The potted willow plant was covered with a TPS bag (500 mm × 350 mm; Mitsubishi Gas Chemical Co.), and closed at the base of its stem with a clip. The TPS bag was fitted using two nozzles. One nozzle was connected to an air cylinder and the other was connected to a glass tube packed with an adsorbent (Tenax TA 60/80, Gerstel GmbH & Co. KG). The glass was connected to an air pump (Sibata Scientific Technology, Ltd.). Purified air from the cylinder was directed into the TPS bag, and volatile compounds from the headspace of the plant were collected in the adsorbent by generating an air flow (100 mL/min) using an air pump collected on the adsorbent for 40 min.

The collected volatile compounds were analyzed using a gas chromatograph mass spectrometer (6890/5972 GC–MS (mass selective detector, 70 eV), Agilent Technologies, Santa Clara, CA, USA) equipped with an HP‐5MS capillary column (Agilent; length 30 m, ID 0.25 mm, film thickness 0.25 mm). The system was equipped with a thermal desorption system (TDS), cooled injection system, and cold trap system (CTS, GERSTEL GmbH & Co. KG). Headspace volatiles collected on the adsorbent were released by heating the TDS at 280°C for 4 min. Flash heating of the cold trap unit induced sharp injection of the compounds into the capillary column of the GC. The GC oven temperature was programmed to increase from 40°C (9‐min hold) to 280°C at a rate of 5°C/min. The headspace volatiles were tentatively identified by comparing their mass spectra with those from Wiley databases (Wiley7N and Wiley275) and the database of the National Institute of Advanced Industrial Science and Technology (SDBS compounds and spectral search; http://riodb01.ibase.aist.go.jp/sdbs/cgi‐bin/direct_frame_top.cgi). The retention times of the volatiles were further compared with those of standard compounds (Wako Chemicals, and IFF Chemical). Compounds for which no standards were available were regarded as tentatively identified when >90% of spectra matched those in the databases.

### 2.3 Community monitoring

The number of arthropods found on each plant was surveyed from Day 1 when the potted willow plants were placed in a common garden and was observed for 9 days (at 9 AM, 12 PM, and 3 PM on Day 1–2, and at 9 AM and 3 PM from Day 3–8 and at 9 AM at Day 9 (June 29, 2016)).

### 2.4 Deer herbivory

The shoots of potted willow plants were found to be consumed by deer (*Cervus nippon*) early in the morning before the survey on June 23 (Day 3 of the experiment). The potted willow damaged with deer herbivory was recorded (11 pots in total: Table A1). After that, a plastic net with a 10‐cm mesh was covered around a plot where potted willow plants were placed (1 m away from the outermost pots). A trail camera was installed around the experimental plot in the common garden, and photos captured deer walking around the willow garden early morning on June 24, indicating that the damage on the night of Day 2 was likely due to deer herbivory.

### 2.5 Statistical analysis

#### 2.5.1 Data preprocessing

All statistical analyses and visualizations were conducted using R4.2.0 (R Core Team, 2022). The raw datasets of community monitoring for 9 days with multiple measurements within a day were first separated into each day subsets. On this short‐term scale, it is reasonable to assume that mortality, immigration, and emigration may play a larger role in arthropod population dynamics than local birth processes on each plant. In addition, the appearance of an arthropod individual at each measurement time point could be just a transient stay on the plant. For each of the colonizer communities from Day 1 to Day 9, the cumulative number of individuals observed for each species was calculated to reflect the persistence of each individual stay and regarded them as community compositions at each day. We calculated the 2‐day moving sum of the abundance of each observed species (i.e., combining data from consecutive days, such as Days 1 and 2, Days 2 and 3, up to Days 8 and 9, for the colonizer communities), in order to reduce sparseness in the community compositions. These two types of communities (communities of each day and those at 2‐day scale) were used for further statistical analysis. For the analysis of temporal dynamics of populations and communities, we primarily utilized population size and community composition data of each day. To compare the differences between the beginning and end of the 9‐day time series, we focused on the earliest colonizer community (Days 1–2 community) and the subsequent colonizer community (Days 8–9 community).

#### 2.5.2 Basic analysis of populations and communities

Prior to applying multivariate statistics to the community compositions, we summarized the temporal dynamics of key parameters in the communities, including the total abundance of colonizers, taxonomic richness, and population sizes of major taxa. We generated the rank abundance curve by cumulatively adding up the abundance of each taxon and then selected the top five taxa to examine their population dynamics. We used the gls function in the nlme package, which incorporates an autoregressive structure of order one (i.e., AR(1)), for a repeated measures ANOVA to examine the relationship between explanatory variables (plant species identity, deer herbivory, PVOCs, and time; See also Section 2.5.5) and response variables (the total abundance of colonizers, taxonomic richness, and population sizes of major taxa).

#### 2.5.3 Distance‐based approaches

The zero‐adjusted Bray–Curtis dissimilarity for community composition, which is more appropriate than Bray–Curtis dissimilarity for sparse samples in the initial phases of community establishment (Clarke et al., 2006), and the Hellinger dissimilarity for PVOC composition were used to convert the raw composition data into distance matrices. To confirm whether the presence/absence patterns of each taxon differed between samples, we first binarized the composition matrix and then applied the zero‐adjusted Bray–Curtis dissimilarity, since Bray–Curtis dissimilarities are influenced by differences in abundances even when the presence/absence patterns are identical.

When examining the temporal changes in community composition on each plant individual, we calculated the dissimilarities between the community compositions at each day using two methods. The first method involved calculating the dissimilarities between the community composition on Day 1 and on each day thereafter (from Day 2 to Day 9). The second method involved comparing the communities at each day with those on the previous day to calculate the dissimilarities between neighboring day communities. Since raw temporal series of compositional data were converted into temporal differences, we did not consider AR(1) structure and simply applied linear model to these datasets when investigating the influences of plant species identity and deer herbivory. More specifically, we first prepared the full model (divergence ~ days + plant/plant clone + deer_arrival + days: plant + days:deer_arrival + plant:deer_arrival) and then selected the best model by stepAIC function.

When testing whether each of these compositions (i.e., PVOC composition, earliest colonizer community, and subsequent colonizer community) differed between categorical parameters (e.g., plant species identity and the presence/absence of deer herbivory), PERMANOVA (vegan::adonis) was employed after checking for the absence of heterogeneity of divergence (dispersion) within a level through PERMDISP (vegan::betadisper), with 4999 permutations. This is the same when checking if the community composition differed between days only when homogeneous dispersions between days were confirmed. Note that we also investigate the relationship between deer herbivory (occurring at the night of day 2), PVOC composition (evaluated before the experiment), and earliest colonizer community (observed in day 1–2) in order to confirm whether deer herbivory occurred randomly or was related to plant traits or conditions. The Mantel test (vegan::mantel) was also used to test for the presence of an overall linkage between two distance matrices.

Plant volatile organic compound composition in our study represents one of the constitutive plant traits since PVOCs were collected from plants without any herbivory. In order to quantify the degree of the inducibility of PVOCs, we also used the PVOC composition data from our previous study (Yoneya et al., 2012). We collected PVOCs from four replicates of each species intact individuals without herbivory and those infested with leaf beetle (*Plagiodera versicolora*). The dissimilarity between PVOCs was calculated by the Hellinger distance as the same as our main analyses and the spatial median of replicates was obtained by using *betadisper*() function in *vegan* package of R. Distance between the spatial median of the intact individuals and that of the infested individuals represents the inducibiliy of PVOCs for each willow species. Note that the source plant individuals for this PVOCs analysis were not identical to those in our study but originated from the same population (see Yoneya et al. (2012) for more details of PVOC collection).

#### 2.5.4 Raw‐based approaches

We relied on Procrustes analysis, following the standard procedure for ecological datasets (Lisboa et al., 2014; Peres‐Neto & Jackson, 2001). We directly compared the linkage between two raw matrices (vegan::procrustes) using the first two or five axes of principal coordinate analysis (PCoA) and checked its statistical significance with 4999 permutations (vegan::protest). This Procrustes test is comparable with Mantel test but has greater power for detecting the linkage between matrices (Peres‐Neto & Jackson, 2001). We also used the Procrustes residual value (univariate) as an indicator of the deviation (or Procrustean association metric, Lisboa et al., 2014) of each observation (each plant individual in this study) from the overall linkage between two matrices, for further analysis of variance (ANOVA). To investigate the relationship between the first and final 2‐day communities (i.e., the earliest colonizer community vs. the subsequent colonizer community), we also used partial Procrustes analysis to remove the effects of PVOCs on these communities. We constructed three matrices from the PCoA scores of PVOCs, the earliest colonizer communities, and the subsequent colonizer communities, and then applied the partial Procrustes analysis to these matrices. Since the existing function for partial Procrustes analysis (SINCSA::procrustes. partial) only returns the partial Procrustes correlation but does not the Procrustes residual values, we created our own custom function. This first uses *res_cal* function in *gustave* package to calculate the residuals of the community matrices after regressing them against the PCoA matrix and then uses these residuals as the input of *procrustes* function in *vegan* package.

#### 2.5.5 Overview of statistical variables

The statistical analyses in our study incorporated various response and explanatory variables, each serving a specific purpose. In the linear model for basic analysis of populations and communities, the response variables were continuous univariates (total abundance, taxonomic richness of arthropods, and population size of abundant taxa), while the explanatory variables consisted of categorical variables (plant species identity, plant clones, and deer herbivory) and continuous univariates (the first two axes of PCoA of PVOC composition and time). PERMDISP and PERMANOVA utilized multivariate continuous variables (dissimilarity matrices from PVOC composition and arthropod community composition) as response variables, and the explanatory variables are categorical only (plant species identity, plant clones, and deer herbivory). Time (day) specifically acted as a categorical explanatory variable for analyzing time‐dependent arthropod community compositions (PERMDISP) but not used for assessing the snapshot of composition (i.e., the earliest and subsequent communities). When examining temporal changes in community compositions, the linear model was employed with univariate continuous variables (dissimilarity of community composition over time) as response variables and categorical variables (plant species identity, plant clones, and deer herbivory) and continuous variable (time) as explanatory variables. Both Mantel test and Procrustes test utilized multivariate dissimilarity matrices from PVOC composition and arthropod community composition as both response and explanatory variables. In ANOVA for Procrustes residuals, the response variables were continuous univariates (Procrustes residuals), while the explanatory variables were categorical (plant species identity, plant clones, and deer herbivory). Notably, as this ANOVA was unrelated to time, the explanatory variables did not include time. In all analyses except for PERMDISP, plant clones were treated as a nested factor within plant species identity.

#### 2.5.6 Data visualization

Both the base plot functions and ggplot2 packages were used. More specifically, PCoA (vegan::capscale) and cluster dendrogram (vegan:hclust) for two‐dimensional nonhierarchical clustering and hierarchical clustering were used, respectively.

## 3 RESULTS

### 3.1 Observed diversity of PVOCs and arthropods

Twenty‐eight VOCs in total were identified from the headspace around each potted plant (e.g., (*Z*)‐3‐Hexenyl acetate, (*E*)‐4,8‐dimethyl‐1,3,7‐nonatriene, (*E*)‐beta‐ocimene); the list of VOCs is available online (https://dx.doi.org/10.6084/m9.figshare.23583426). Moreover, 19 arthropod species/group (12 herbivores, seven predators/parasitoids) and one snail species (noting that a single individual was observed only once, although it was not an arthropod but was included in the community composition) were identified (Table 1).

**TABLE 1:** List of groups of plant users (20 arthropod groups and a snale).

Group of plant users	Information of group	Functional group
leaf_beetle1	*Plagiodera versicolora*	herbivore
leaf_beetle2	*Anomala orientalis*	herbivore
leaf_beetle3	the other leaf beetles	herbivore
grasshopper	grasshoppers	herbivore
lepidoptera	lepidopteras	herbivore
snale	a snale	herbivore
aphid1	*Chaitophorus saliniger*	herbivore
aphid2	*Aphis farinosa*	herbivore
treehopper	treehoppers	herbivore
flower_bug	Anthocoridae	herbivore
lace_bug	*Metasalis populi*	herbivore
green_lacewing	Chrysopidae	predator
sawfly	unidentified	herbivore
parasitoid1	unidentified	parasitoid
parasitoid2	unidentified parasitoid of aphid	parasitoid
parasitoid3	unidentified parasitoid of *P. versicolora*	parasitoid
ladybeetle	*Coccinella septempunctata*	predator
hover_fly	Syrphidae	predator
Thrips	*Thrips* sp.	herbivore
Mantis	*Mantis* sp.	predator
spider	spiders	predator

### 3.2 Basic analysis of populations and communities

**FIGURE A1:**
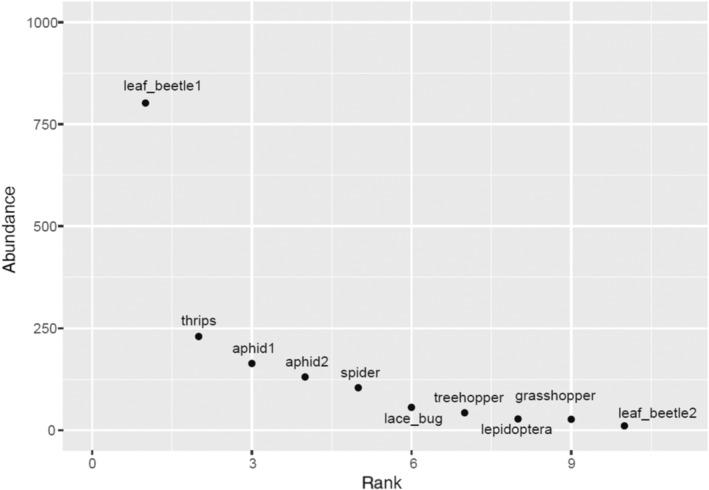
Rank abundance curve of plant users. The top 10 taxa are presented based on the cumulative abundance in all of the plant individuals.

**FIGURE A2:**
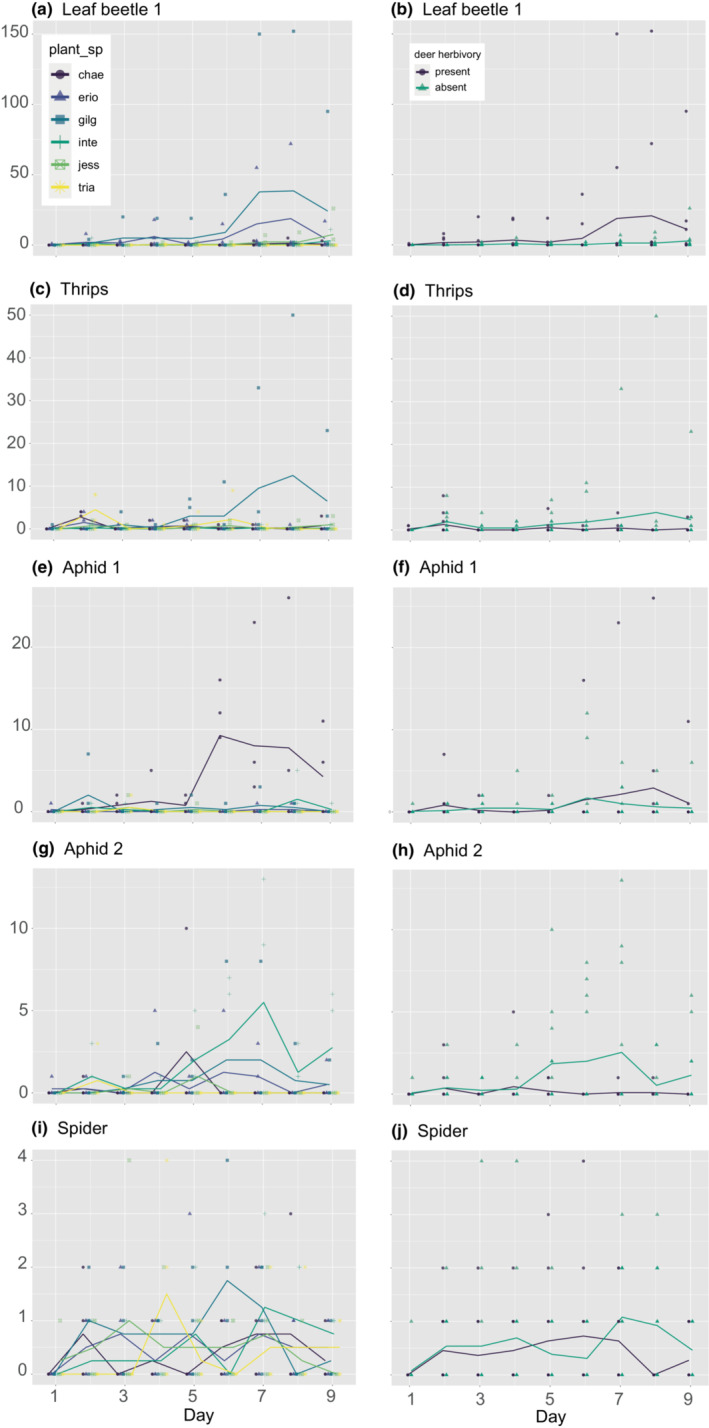
Population dynamics of five most abundant taxa. Each dot represents the abundance on each plant individual while the lines are the averages for each plant species (a, c, e, g, i) and presence/absence of deer herbivory (b, d, f, g, j).

Based on the rank abundance curve (Figure A1), we identified top five abundance taxa: leaf_beetle1 (*Plagiodera versicolora*), thrips (thrips sp.), aphid1 (*Chaitophorus saliniger*), aphid2 (*Aphis farinosa*), and spider (spider). Differences in their population dynamics between plant individuals were explained by plant species identity for two aphid species (*C. saliniger* and *A. farinosa*), by plant clones for two aphid species and spider, and by deer herbivory for *A. farinosa* (Figure A2; Table A2).

**FIGURE 1:**
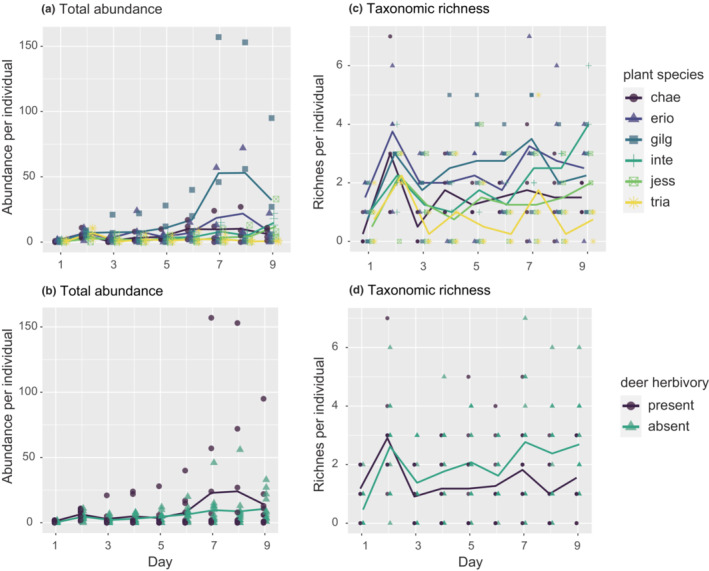
Temporal development of total abundance and taxonomic richness. Total abundance (a, b) and the taxonomic richness (c, d) depending on plant species and the presence/absence of deer herbivory, respectively.

Differences in the total abundance of colonizers in the communities were not explained by plant species identity (df = 5, *F* = 0.943, *p* = .4542), plant clones (df = 6, *F* = 0.633, *p* = .704), and deer herbivory (df = 1, *F* = 0.427, *p* = .514) while those in taxonomic richness were explained by plant species identity (df = 5, *F* = 9.7828, *p* < .0001), plant clones (df = 6, *F* = 7.127, *p* < .001), and deer herbivory (df = 1, *F* = 8.4426, *p* = .0041, interaction with day df = 1, *F* = 10.6293, *p* = .0013; Figure 1). In particular, taxonomic richness on *S. eriocarpa* (**Erio**) was greater than on S. *chaenomeloides* (**Chae**; Figure 1c, *p* = .0037).

In addition, taxonomic richness was greater on plant individuals with deer herbivory at the 1st day (post hoc linear model *p* = .023) but became smaller than plant individuals without herbivory after deer arrival at the 2nd day night (Figure 1d).

### 3.3 Distance‐based approach

**FIGURE 2:**
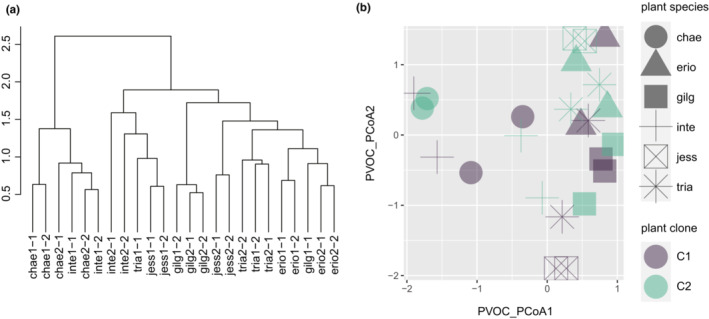
PVOC composition depending on plant species identity and plant clones. (a) Hierarchical cluster tree to clarify the clustering of two plant individuals from the identical intraspecific clones. (b) PCoA plots to clarify the entire compositional differences. The first two axes of PcoA plot explained 23.8% and 13.79% variations, respectively.

Variations in PVOC composition between individual plants were explained by plant species identity (nested PERMANOVA, df = 5, *F* = 4.005, *p* = .001) but could not be evaluated for plant clones due to heterogeneous dispersion (PERMDISP, df = 11, *F* = 9.458 × 10^29^, *p* = .001; Figure 2). The first two axes of PCoA of PVOC composition were also used in the linear model for the basic analysis of populations and communities but there were no statistically significant associations with five abundant taxa, total abundance, and taxonomic richness (*p* > .05).

**FIGURE A3:**
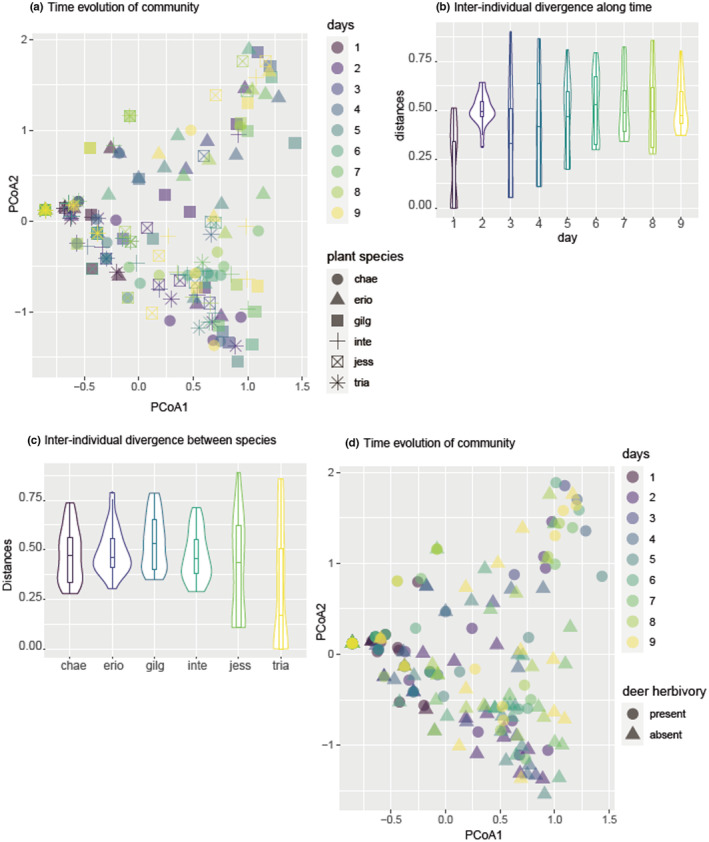
Time evolution of community in a single day scale (a) PCoA Plot for community composition at each day. (b) Interindividual divergence along time. The dispersions within group were statistically heterogeneous (PERMDISP, df = 5, *F* = 8.0384, *p* = .001). The R codes for this analysis is available in the section [6. 3.2] of the Rnotebook html.

**FIGURE A4:**
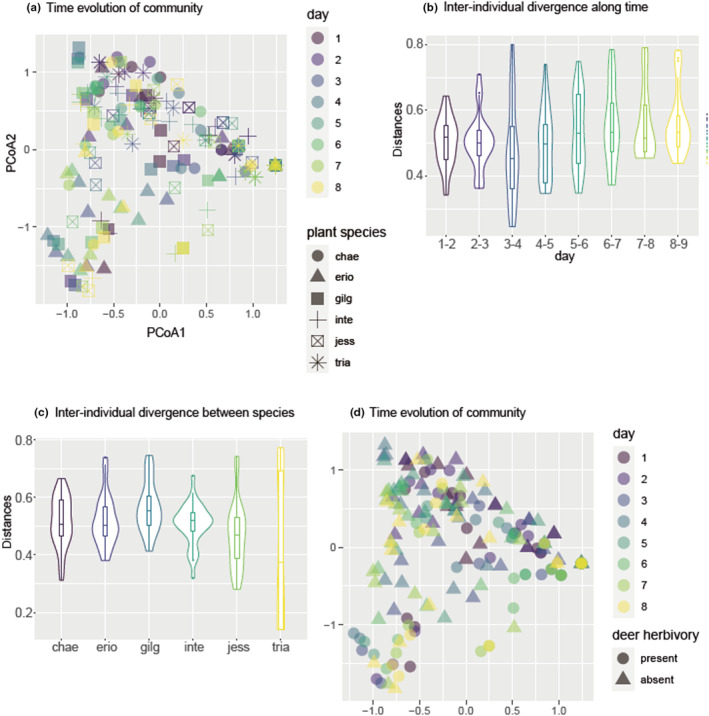
Time evolution of community in 2‐day scale (a) PCoA Plot for community composition at each day. (b) Interindividual divergence along time. The statistical results were presented in the main text. (c) Inter‐individual divergence between plant species. The R codes for this analysis is available in the section [6. 2.2] of the Rnotebook html.

The community development patterns in 1‐ and 2‐day scales were not visually captured by simple PCoA plots (Figures A3 and A4). Interindividual divergences in community composition depended on time (PERMDISP, df = 8, *F* = 5.86, *p* = .001 for each day community and df = 7, *F* = 2.2591, *p* = .032 for 2‐day community), plant species (df = 5, *F* = 8.0384, *p* = .001 for each day community and df = 5, *F* = 4.0995, *p* = .005 for 2‐day community), and plant clones (df = 11, *F* = 5.8215, *p* = .0001 for each day community and *F* = 3.9881, *p* = .001 for 2‐day community), but not on deer herbivory (df = 1, *F* = 1.7701, *p* = .189 for each day community and *F* = 0.1087, *p* = .776 for 2‐day community). Due to these heterogeneous dispersions, it was not possible to evaluate the differences of community composition along time, between plant species, and between plant clones by PERMANOVA. Although PERMANOVA demonstrated the statistically significant differences of community composition with or without deer herbivory (df = 1, *F* = 5.3838, *p* = 4.0 × 10^−4^ for each day community and *F* = 7.4711, *p* = 2.0 × 10^−4^ for 2‐day community), these were not simply captured by PCoA plots (Figures A3d and A4d).

**FIGURE 3:**
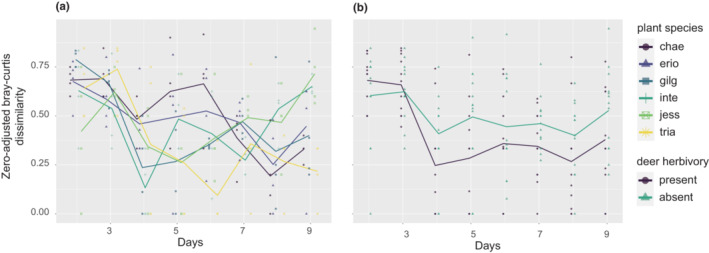
Divergence of community along time on each plant individual. Zero‐adjusted Bray–Curtis dissimilarity between communities in each day and those in the previous day, depending on plant species (a) and the presence/absence of deer herbivory (b).

**FIGURE A5:**
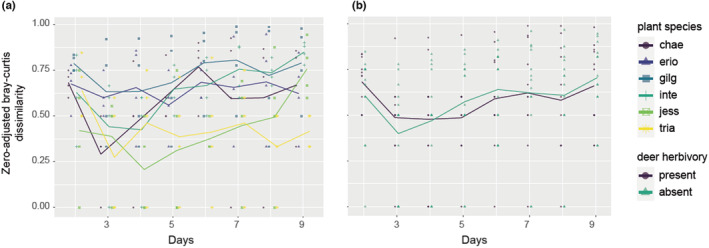
Divergence of community along time on each plant individual. Zero‐adjusted Bray–Curtis dissimilarity between communities in each day and those in the first day, depending on plant species (a) and the presence/absence of deer herbivory (b). The R codes for this analysis is available in the section [6. 3. 4] of the Rnotebook html.

The development of colonizer community was better captured with day‐by‐day shifts in community composition evaluated on each plant individual (Figure 3).The best model included days, plant species identity, deer herbivory, plant clones, and the interaction between days and plant species identity (Table A3). The divergence between neighboring days decreased with day (linear model, df = 1, *F* = 17.4, *p* = 2.46 × 10^−5^), and its dependence on plant species identity changed with day (df = 5, *F* = 4.4698, *p* = .0007429; Figure 3a). In addition, the divergence was lower when deer herbivory was present (df = 1, *F* = 5. 4493, *p* = .020724; Figure 3b). The similar patterns were obtained when evaluating the divergence between 1st day community and each day community (Figure A5; Table A3).

**FIGURE 4:**
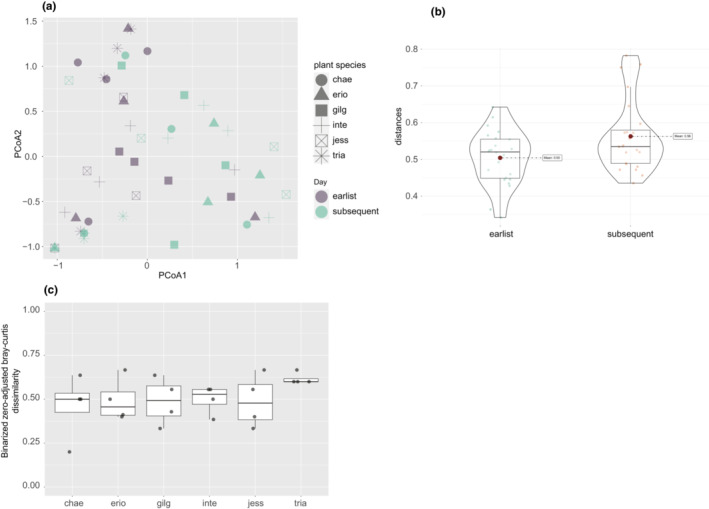
Earliest and subsequent colonizer communities. (a) PCoA plot to visually confirm that two communities along time development were not identical. The first two axes of PcoA plot explained 18.3% and 15.1% variations, respectively. (b) Interindividual divergence of arthropod communities, depending on the day (1st and 2nd vs. 8th and 9th). (c) Differences in taxonomic composition between earliest and subsequent communities on each plant individual, evaluated by zero‐adjusted Bray–Curtis dissimilarity of binarized taxonomic abundance data.

**FIGURE A6:**
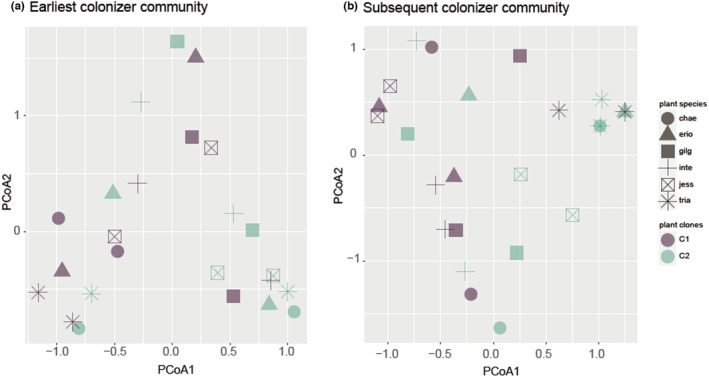
Separate PCoA ordination for earliest and subsequent colonizer community (a) PCoA for the earliest colonizer community at Day 1–2. (b) PCoA for the subsequent colonizer community at Days 8–9. The R codes for this analysis is available in the sections [6.4.2] & [6.4.3] of the Rnotebook html.

The difference between the centroid positions of earliest colonizer community and subsequent colonizer community was not clear (Figure 4a) because of heterogenous dispersions; that is, interindividual divergence of the composition was significantly greater in the subsequent colonizer community (Figure 4b, PERMDISP, df = 1, *F* = 5.4944, *p* = .029). However, on each plant individual, the taxonomic composition did change (Figure 4c). The earliest colonizer community (Figure A6a) was not clustered by plant species identity (nested PERMANOVA, df = 5, *F* = 0.9983, *p* = .4756), plant clone (df = 6, *F* = 0.7222, *p* = .9072), or deer arrival (df = 1, *F* = 0.8917, *p* = .5368). Similarly, the subsequent colonizer community (Figure A6b) was not clustered by plant species identity (nested PERMANOVA; df = 5, *F* = 1.3892, *p* = .0884) or plant clones (df = 6, *F* = 1.2118, *p* = .2856). There was also no influence of deer herbivory (PERMANOVA, df = 1, *F* = 1.3536, *p* = .196). The overall correlation evaluated using the Mantel test was significant (*r* = .178, *p* = .009) for the earliest colonizer community versus the subsequent colonizer community but was not significant for PVOCs versus the earliest colonizer community (*r* = −.01594, *p* = .5756), and PVOCs versus the subsequent colonizer community (*r* = .03793, *p* = .3088), respectively.

### 3.4 Raw‐based approaches

**FIGURE 5:**
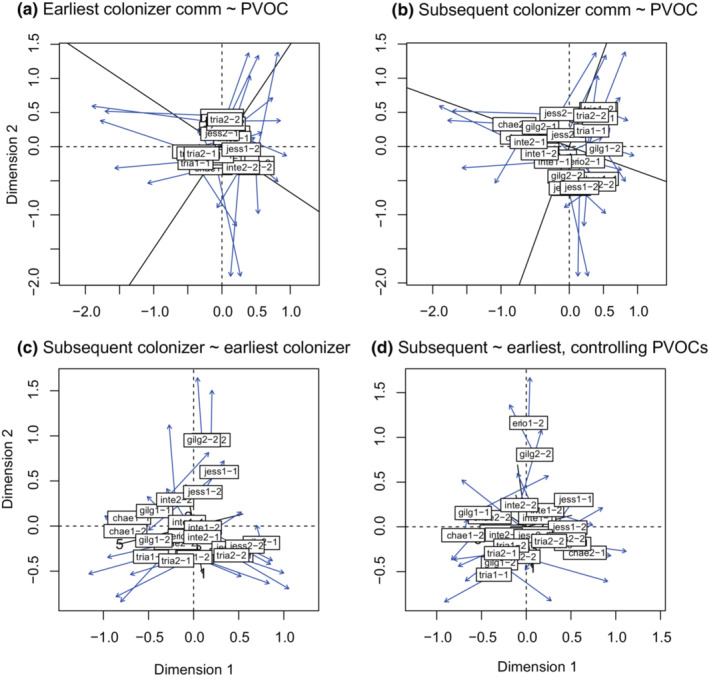
Procrustes residuals distribution. The ordination of the response variables (Y matrix) was rotated to minimize the squared difference with the ordination of the explanatory variables (X matrix). The matrix with the boxed texts is the rotated Y matrix. This represents the component of Y that is explained by X, and the endpoint of the blue arrows represents the corresponding point of the X matrix. (a) X and Y are PVOCs and the earliest colonizer community, respectively. (b) X and Y are PVOCs and the subsequent colonizer community, respectively. (c) X and Y are the earliest colonizer community and the subsequent colonizer community, respectively. (d) Linear relationships between communities and PVOCs was first partial out and then X and Y are set as the residuals of the linear regression with PVOCs of the earliest colonizer community and those of the subsequent colonizer community, respectively.

**FIGURE 6:**
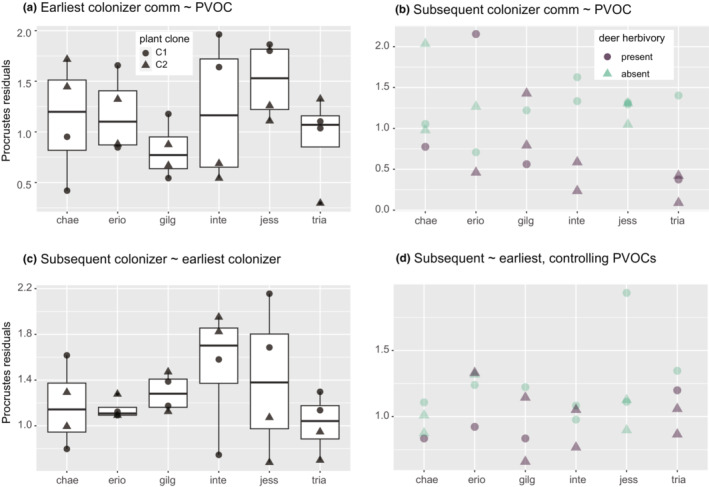
Procrustes residuals depending on other factors. The residuals of the linkage between two matrices (Procrustes correlation: Y ~ X) can be grouped by other factors (i.e., the plant species identity and deer herbivory). (a) Residuals of the linkage of the earliest colonizer community from PVOCs, depending on plant species identity. (b) Residuals of the linkage of the subsequent colonizer community from PVOC, depending on plant species identity and deer herbivory. (c) Residuals of the linkage of the subsequent colonizer community from the earliest colonizer community. (d) Residuals of the linkage of the subsequent colonizer community from the earliest colonizer community, after partialling out the PVOCs influence. The grouped residuals are presented by box plots with raw data (dots) (a and c) or by raw data (dots) (b and d).

The earliest colonizer community was not explained by PVOCs (symmetric Procrustes rotation correlation: *r* = .2767, *p* = .3308; Figure 5a). The linkage strength (although not significant) was not explained by plant species identity (nested ANOVA df = 5, *F* = 1. 9116, *p* = .16596) but clustered by plant clones (df = 6, *F* = 3.8013, *p* = .02342; Figure 6a). However, the subsequent colonizer community was explained by both PVOCs (symmetric Procrustes rotation correlation: *r* = .4354, *p* = .0198; Figure 5b) and the earliest colonizer community (symmetric Procrustes rotation correlation: *r* = .5374, *p* = .0036; Figure 5c). Interestingly, the linkage strength between the subsequent colonizer community and PVOCs was stronger (Procrustes errors (Figure 5b) were smaller) for plant individuals with deer herbivory (ANOVA, df = 1, *F* = 8.645, *p* = .007571; Figure 6b), whereas the variations in the linkage strength were not explained by plant species identity (nested ANOVA, df = 5, *F* = 0. 9508, *p* = .4839) or plant clones (df = 6, *F* = 1.4679, *p* = .2687). On the contrary, the linkage strength between the subsequent and earliest colonizer communities was not explained by plant species identity (df = 5, *F* = 1.4537, *p* = .28052) and deer herbivory (df = 1, *F* = 0.1113, *p* = .74491) but clustered by plant clones (df = 6, *F* = 3.2848, *p* = .04197; Figure 6c).

After partialling out the influence of PVOCs on the earliest and subsequent colonizer communities, a significant correlation between these two communities still remained (Figure 5d, Procrustes rotation correlation: *r* = .5781, *p* = 4.0 × 10^−4^). The relationship between the two communities was not explained by plant species identify (df = 5, *F* = 1.3372, *p* = .31861) and plant clones (df = 6, *F* = 1.1802, *p* = .38320). Additionally, the relationship appeared to be stronger on plant individuals that experienced deer herbivory (Figure 6d), although the difference was not statistically significant (*p* = .09582).

## 4 DISCUSSION

### 4.1 Impact of PVOCs on community assembly

PVOCs were an effective predictor of overall arthropod community assembly, as suggested by Procrustes analysis. Although the Mantel test did not detect the linkage between PVOCs and communities, we assume that Procrusted analysis have a greater statistical power (Peres‐Neto & Jackson, 2001). Despite the pattern that PVOCs were plant species‐specific, plant species identity itself explained a more limited range of community characteristics, such as population dynamics of a few abundant taxa, taxonomic richness, and the temporal development of the community. The species specificity of PVOCs described in this study (Figure 2) is consistent with recent studies on willows (Yoneya et al., 2012) as well as vegetable species (Yoneya et al., 2018). Similar to the genotype specificity of other plant traits (e.g., phenolics) of willow (Lehrman et al., 2012), willow PVOCs were also genotype‐specific. Thus, the PVOCs of uninfested plants can be interpreted as a major constitutive plant trait and act as an environmental filter for community assembly. However, the community assembly pattern was not explained by plant species identity because of substantial intraspecific (i.e., between clones and within a clone) variations in PVOC composition. Although there was an overall correlation between PVOCs dissimilarity and the subsequent colonizer community dissimilarity, very similar PVOCs composition of the replicates from the identical clone did not always result in very similar subsequent colonizer community (e.g., *Salix serissaefolia* (**Jess**), Figure 2b vs. Figure A6b).

Our results indicated that the environmental filtering function of PVOCs did not immediately become apparent when arthropods were allowed to colonize intact plant individuals. The effects of PVOCs as attractants for arthropod immigrations are weak and complicated by the stochastic behavior of organisms in very short term (within 2 days). However, PVOCs would influence the colonizer's choice of whether to stay on the plant individual, and the gradual increase in taxonomic richness and abundance on an individual plant made the link between PVOCs and community composition more apparent (within 9 days). This could explain why the link between PVOCs and community assembly was statistically significant for the subsequent colonizer community, but not for the earliest colonizer community.

**FIGURE 7:**
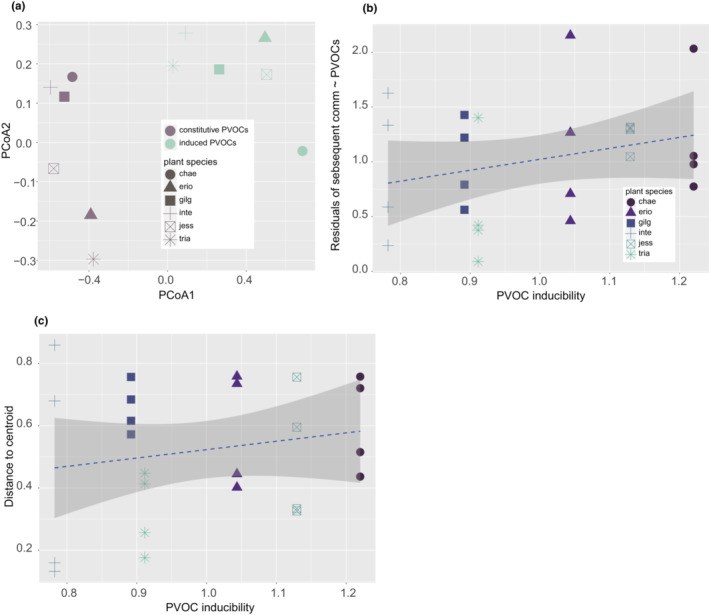
Inducibility of PVOCs and relationships with community assembly. (a) Spatial median distribution of four replicates within each plant species for constitutive and induced PVOCs (infested with leaf beetle, *Plagiodera versicolora*), respectively. (b) The relationship between the inducibility of induced PVOCs (distance between spatial medians of constitutive and induced PVOCs for each species) and the Procrustes residual of (constitutive) PVOCs (Figure 5b) fitted by the subsequent colonizer community. (c) The relationship between the inducibility of induced PVOCs and interindividual divergence within plant species (Figure A6b) for the subsequent colonizer community.

Although not statistically significant, the linkage between PVOCs and the subsequent colonizer community was highly variable among plant species (Figure 6a). PVOCs in our experiment can be interpreted as one of the constitutive traits of plants because they were collected and quantified from intact (uninfested) plant individuals. One would hypothesize that plant species with greater inducibility to herbivory shift PVOC composition after herbivory to a greater extent, which in turn affects the subsequent assembly, weakens the linkage between the constitutive PVOCs and community composition, and leads to a greater inter‐individual divergence of community composition. In fact, when re‐analyzing the data from a previous study (Yoneya et al., 2012), there were interspecific variations in the degree of dissimilarity between constitutive PVOCs and leaf beetle‐induced PVOCs (Figure 7a). However, there were no linkages with Procrustes errors (Figure 7b, linear regression, *p* = .178, adjusted *R* ^2^ = 0.039) and with interindividual divergence (Figure 7c, linear regression, *p* = .375, adjusted *R* ^2^ < 0). Although accumulating evidence suggests that induced PVOCs act as attractants and repellants for predatory and parasitoid arthropods as well as herbivorous arthropods (Beyaert & Hilker, 2014; Yoneya et al., 2010, 2012); it remains unclear whether induced PVOCs are important determinants of arthropod community assembly.

### 4.2 Roles of unmeasured plant traits in community assembly

There is accumulating evidence suggesting the importance of chemical and physical defense in structuring arthropod communities. Specifically, the composition of secondary metabolites and physical defense levels are species‐specific constitutive traits in Salicaceae species, which are linked to herbivore composition (Volf et al., 2015). However, such unmeasured plant species‐specific constitutive traits were unlikely to influence the earliest and subsequent colonizer communities, since these communities were not clustered by plant species identity in this study. Similarly, the lack of linkage between the subsequent colonizer community and plant species identity implies that plant species‐specific induced defense was unlikely to influence the community assembly. Induced defense also depends on the types of herbivory (chewer vs. sucker), and is related to priority effects (Stam et al., 2018). *P. versicolora*, common herbivore in Salicaceae species, initiates the jasmonic acid pathway (Liu et al., 2022) and would negatively affect the late emerging conspecifics' performance (Yoneya et al., 2014) and its population dynamics (Yoneya et al., 2023). Additionally, at the interannual scale, willow species in floodplain often show compensatory regrowth after herbivory and physical damages, leading to greater resource availability and better nutritious quality (e.g., lower C:N ratio) for herbivores (Nakamura et al., 2005, 2006). These herbivory‐specific induced traits may have depended on the composition of the earliest colonizer community. Therefore, the presence of a linkage between the earliest and subsequent colonizer communities, even after controlling for the influences of PVOCs, suggests a potential role for these induced traits in shaping the subsequent colonizer community, despite not being measured in our study. In contrast, the earliest colonizer community was more likely to be structured stochastically, or theoretically influenced by unmeasured traits that are not plant species‐specific nor PVOC‐correlated.

### 4.3 Priority effect

Our results suggest that both the priority effect and environmental filtering play significant roles in the observed community assembly processes. Linkage between the earliest and subsequent colonizer communities suggests the presence of priority effects caused by plant–arthropod feedback (Utsumi et al., 2010; Yoneya et al., 2023). One may suspect that the high linkage from the Procrustes analysis was obtained simply because the community composition did not change in such a short period. However, this is unlikely because the presence/absence patterns of species changed on all plant individuals (Figure 4c). Another possible explanation for the linkage between the earliest and subsequent colonizer communities is that they shared a common influence of PVOCs. Even after controlling for the influence of PVOCs, a significant correlation between the earliest and subsequent colonizer communities remained, suggesting the presence of priority effects. However, the specific mechanisms behind this effect, including whether indirect interspecific interactions mediated by induced plant traits can occur in such a short time remains unexplored.

A priority effect is stronger with a stronger “herbivore specificity” of the herbivore‐induced plant traits, that is, when the dependence of herbivore‐induced plant traits on herbivore species identity is stronger. One may hypothesize that the inducibility of PVOCs shown in Figure 7a can be used as a loose proxy, but it is not. While the inducibility of PVOCs represents the differences between constitutive PVOCs and those induced by a single herbivore species (*Plagiodera versicolora*), herbivore specificity should be evaluated using the differences in the induced traits between those induced by different herbivore species. The linkage strength between the earliest and subsequent colonizer communities was not associated with the inducibility of PVOCs (linear regression, *p* = 0.5383, adjusted *R* ^2^ < 0).

### 4.4 Impact of deer herbivory

Unexpected deer herbivory also had a substantial effect on arthropod community assembly. The counterintuitive finding that plant individuals with deer herbivory had a stronger link between PVOC and the subsequent colonizer community on Days 8 and 9 (Figure 6b) could be explained by the suppressive effects of deer herbivory on taxonomic richness (Figure 1b) and the temporal changes in community composition (Figure 3b). The deer herbivory may have short‐term negative impacts on resource availability, but such suppression occurred without negative impacts on the total abundance of arthropods (Figure 1a) and four major taxon groups (except for aphid2; Figure A2). This suggests that the decline of architectural complexity, such as branching structure complexity, might hinder the colonization of new species (Marquis et al., 2002; Rudgers & Whitney, 2006), once the community composition was determined by the preference to PVOCs.

Mammals (e.g., elephant and wallaby) as well as arthropods use PVOCs as foraging cues (McArthur et al., 2019; Stutz et al., 2016). In light of this, the associations between deer herbivory, PVOCs, and arthropod communities may also imply the effects of PVOCs and arthropod communities on deer behavior. However, PVOCs measured before the assembly experiment, which represent constitutive plant traits, were not grouped by the presence/absence of deer herbivory (PERMANOVA, df = 1, *F* = 1.6856, *p* = .0686). Since the earliest colonizer communities were not grouped by the presence/absence of deer herbivory, it is unlikely that plant traits were modified by the earliest colonizers and then attracted deer. Notably, we observed a higher taxonomic richness on plants at the 1st day that would later be subjected to deer herbivory at night on the 2nd day (Figure 1). This observation, however, remains inconclusive because our experiment was not intended to test the impact of plant conditions on deer behavior. Therefore, we assumed that the arrival of deer occurred as a stochastic event, while the community assembly after deer arrival (during the night of Day 2 of the experiment) was affected by deer herbivory.

Long‐term (months to years) studies suggest that deer herbivory can induce changes in plant physical and chemical traits (Bryant, 2003; Den Herder et al., 2004; Stephan et al., 2017; Tanaka & Nakamura, 2015), but it remains unclear what can happen in the short term (9 days). To the best of our knowledge, there have been no studies on mammal‐induced PVOCs. However, it is likely that deer herbivory induced changes in PVOCs during our experiment, because the induction of PVOCs after herbivory or mechanical damage typically occurs on a very short timescale (starting within minutes and hours and continuing for several days; Loreto et al., 2006; Martin et al., 2003; Staudt & Lhoutellier, 2007). Deer herbivory destroys a greater part of plant tissues at once than arthropod herbivory, and its impact on plants would be similar to mechanical damage, which is known to increase the quantity as compensatory regrowth, alter the composition of PVOCs, and maintain species specificity (Peacock et al., 2001). We hypothesized that deer herbivory increased PVOC emissions while maintaining species specificity, allowing PVOCs to more effectively serve as behavioral cues for arthropods. This also strengthened the relationship between PVOCs and the subsequent colonizer community by enhancing the environmental filtering roles of constitutive PVOCs in community assembly.

### 4.5 Concluding remarks

In conclusion, our study found that both PVOCs and priority effects played a significant role in the short‐term, initial stages of community assembly of arthropods on willow trees. Although our monitoring was limited to the initial stages, we predict that the effects of community divergence observed in our study was not transient but could persist in a longer timescale, at least for several months, based on previous studies (Stam et al., 2018; Yoneya et al., 2023) indicating such lasting effects. Our study indicated that riparian willow forests are an excellent model system for community assembly, along with recent studies focusing on the local co‐occurrence of multiple willow species (Yoneya et al., 2012), its temporally dynamic characteristics (Nakamura et al., 2005), plant volatile‐mediated food chains (Yoneya & Takabayashi, 2013), and high diversity of arthropods (Nakamura et al., 2006; Yoneya et al., 2023). We would suggest three directions for future research, in addition to longer monitoring of community assembly. First, larger spatial‐scale experiments will elucidate the roles of spatial structure (or dispersal limit; e.g., variation partitioning and spatial clustering, (Coccia & Fariña, 2019)). The distribution of herbivore populations is substantially affected by the diffusibility of PVOCs and the induced responses of locally neighboring plant individuals around damaged trees (i.e., plant–plant communication; Ida et al., 2018; Morrell & Kessler, 2017), which will further influence community assembly. Second, community phylogenetic structure analysis will contribute to evaluate the relative importance of stochastic and deterministic forces in community assembly (Granville et al., 2023; Stegen et al., 2013). Third, herbivore specificities of the induced PVOCs, other defense chemicals, and plant regrowth are key indicators of priority effects via herbivore‐specific‐induced plant traits. These three directions will better predict the relative importance of dispersal limit, environmental filtering, and priority effects in shaping community composition and species diversity on plants.

## AUTHOR CONTRIBUTIONS

**Kinuyo Yoneya:** Conceptualization (lead); formal analysis (supporting); funding acquisition (lead); investigation (lead); methodology (lead); resources (lead); writing – original draft (equal). **Takeshi Miki:** Formal analysis (lead); writing – original draft (equal). **Noboru Katayama:** Investigation (supporting); writing – review and editing (supporting).

## CONFLICT OF INTEREST STATEMENT

The authors declare no competing interests.

## Data Availability

The html generated from the R notebook for basic statistics, all statistical results, and graphics (“VOC_initial_migrator2022_shared.nb.html”) as well as the datasets of PVOCs (“pvoc_en.csv”. “7spp_volatile_rawdata.csv”) and community composition (“grouped_raw_d_en.csv”), which were used in the R notebook, and the list of taxonomy information of arthropods (“arthropod_list.xlsx”) are available in the figshare, DOI: 10.6084/m9.figshare.23583426.

## APPENDIX A

### ADDITIONAL DATA SUMMARY AND STATISTICAL ANALYSES

In this appendix, the additional data summary is provided as tables and the results of additional statistical analyses are presented as figures and tables. The R codes that were used for the analyses in the main text and this appendix are provided as a Rnotebook html with all datasets in the figshare (URL: https://dx.doi.org/10.6084/m9.figshare.23583426).

### SPATIAL DISTRIBUTION OF WILLOW POTS

See Tables A1, A2, A3 and Figures A1, A2, A3, A4, A5, A6

**TABLE A1:** Spatial distribution of the willow pots used for our experiment.

Line 1		Line 2		Line 3	
8	inte1‐2	16	gilg1‐2^a^	24	erio2‐2^a^
7	chae2‐2	15	tria2‐2^a^	23	erio1‐2^a^
6	tria1‐2^a^	14	jess2‐1	22	gilg2‐2^a^
5	chae1‐2^a^	13	inte2‐2^a^	21	jess1‐2
4	erio1‐1	12	jess2‐1	20	tria1‐1
3	jess1‐1	11	erio2‐1	19	chae2‐1
2	inte1‐1	10	gilg2‐1^a^	18	inte2‐1^a^
1	gilg1‐1	9	tria2‐1^a^	17	chae1‐1

^a^ Denotes the plant individuals that were damaged by deer herbivory at the night of Day 2.

**TABLE A2:** Summary of ANOVA for population dynamics.

Factor	numDF	*F* value	*p* value	Factor	numDF	*F* value	*p* value
*Leaf beetle 1 AR1*				*Thrips AR1*			
(Intercept)	1	2.0430437	.1544558	(Intercept)	1	2.898549	.09020526
deer_arrival	1	1.1781529	.2790324	deer_arrival	1	1.214049	.27184939
plant_sp	5	0.5627923	.7284480	plant_sp	5	1.603393	.16072729
day	1	2.574547	.1101653	day	1	1.31827	.25226747
plant_sp:plant_clone	6	0.7250622	.6298854	plant_sp:plant_clone	6	0.651067	.68922852
deer_arrival:day	1	1.1680304	.2811013	deer_arrival:day	1	1.179237	.27881209
*aphid1 AR1*				*aphid2 AR1*			
(Intercept)	1	3.7382613	.05458443	(Intercept)	1	18.886973	2.200889e−05
deer_arrival	1	0.2751856	.60045232	deer_arrival	1	10.04177	1.769259e−03
plant_sp	5	2.4895805	.03256869	plant_sp	5	5.499419	9.077705e−05
day	1	1.558715	.21330571	day	1	3.294054	7.102174e−02
plant_sp:plant_clone	6	0.6049803	.72618265	plant_sp:plant_clone	6	2.514833	2.278461e−02
deer_arrival:day	1	0.4086446	.52338611	deer_arrival:day	1	3.778932	5.329767e−02
*spider AR1*							
(Intercept)	1	60.1859966	4.259846e−13				
deer_arrival	1	1.7404750	1.885795e−01				
plant_sp	5	1.1590098	3.307421e−01				
day	1	2.5012229	1.153302e−01				
plant_sp:plant_clone	6	3.6373696	1.894411e−03				
deer_arrival:day	1	0.9210538	3.383514e−01				

*Note*: The autoregression regarding day was considered before applying anova() function. The main focuses were the effect of plant species identity (plant_sp), plant clones (plant_sp:plant_clone) and presence/absence of deer herbivory (deer_arrival). For this ANOVA table, the linear model did not include the continuous variables (PCoA axes of PVOC composition) as explanatory variables. The R codes for this analysis is available in the section [5.3] of the Rnotebook html.

**TABLE A3:** Summary of ANOVA for Divergence of community along time on each plant individual.

Factor	df	Sum.Sq	Mean.Sq	*F* value	Pr..*F*.
Community divergence day by day					
days	1	0.9204655	0.92046548	18.799340	2.459908e−05
plant	5	0.3859307	0.07718615	1.576429	1.691257e−01
deer_arrival	1	0.2668135	0.26681347	5.449327	2.072442e−02
plant:plant_clone2	6	0.9616737	0.16027895	3.273494	4.485651e−03
days: plant	5	1.0942707	0.21885414	4.469818	7.429398e−04
Residuals	173	8.4705382	0.04896265	NA	NA
Community divergence from Day 1					
days	1	0.3786131	0.37861306	8.889774	3.288981e−03
plant	5	2.5257156	0.50514312	11.860680	7.478112e−10
deer_arrival	1	0.1983031	0.19830312	4.656126	3.234640e−02
plant:plant_clone2	6	2.6237756	0.43729593	10.267639	1.083992e−09
days: plant	5	0.4935816	0.09871632	2.317844	4.558062e−02
plant:deer_arrival	3	0.2905083	0.09683610	2.273696	8.183401e−02
Residuals	170	7.2402535	0.04258973	NA	NA

*Note*: ANOVA was conducted after selecting the best model by stepAIC() function for the full model: divergence ~ days + plant/plant clone + deer_arrival + days: plant + days:deer_arrival + plant:deer_arrival.
